# Paternal Experiences of Perinatal Loss—A Scoping Review

**DOI:** 10.3390/ijerph20064886

**Published:** 2023-03-10

**Authors:** Cecilia Mota, Claudia Sánchez, Jorge Carreño, María Eugenia Gómez

**Affiliations:** 1 Research Coordination in Psychology, National Institute of Perinatology, Mexico City 11000, Mexico; 2 Department of Neurosciences, National Institute of Perinatology, Mexico City 11000, Mexico

**Keywords:** perinatal grief, male grief, psychological impact, scope review

## Abstract

Background: Perinatal grief is one of the most complex and devastating types of mourning for both mothers and fathers; however, there is still little research on the psychological impact on men who experience it. Therefore, the objective of this study was to summarize and synthetize the existing literature on the way men’s grief is experienced. Methods: A search was carried out to examine three databases for articles published in the last four years; 56 articles were obtained, and 12 were retained for analysis. Results: Four common themes were found: the men’s experience of grief, their role as fathers, the impact of the death, and their needs regarding grief and how to face it. Conclusions: There is also a need for discussion of the importance of validating perinatal grief in men and studies that examine it without social gender stigmas in order to provide them with effective emotional support.

## 1. Introduction

Over the years of research in the field of grief caused by the death of a child during the perinatal period, it has been observed that this type of grief is one of the most complex and devastating that a person can experience. It is considered as a traumatic event [1], in which the future parents experience intense grief, and it is not always recognized and accepted by society [2,3]. However, most studies have focused on the impacts that gestational death can have on women, including the measurement of its intensity and the risk factors that can cause them to develop a complicated grief process, while there is still little research on the experience of gestational death, its psychological impact, and the development of grief in men.

In many regions of the world, most of the matters related to pregnancy, childbirth, and parenting were assigned to women until recent times, whereas men are far more involved today [4]. Nevertheless, both maternity and paternity are complex processes that, in many ways, are still influenced by parents’ respective cultures [5]. Various studies have shown that, like mothers, fathers also develop affective bonds with the fetus during the course of a pregnancy [6,7]. These bonds are cultivated by the idea of the unborn child already being part of the family and known by both parents [6]. However, in most contexts, the attention during the gestational period is focused on the maternal role, while the father is rendered invisible by both his social environment and health professionals [8]. 

This situation comes to forefront when parents face perinatal loss. Men generally feel marginalized and misinformed, while their partners receive medical care in health institutions [5]. Regarding the process of perinatal grief, there are studies that indicate that most men experience their grief in a masked way because they feel that they must play a role of strength, understanding, and support for their partners, which prevents them from expressing their own feelings of pain or receiving the necessary psychosocial support [9,10]. Some studies have also noted feelings of anger, guilt [11], and depressive and anxious symptoms [12], as the main emotional manifestations of the perinatal grieving process in men. It is evident that perinatal death has a psychological impact on fathers and can have important consequences on the individual, family, and social spheres; however, when compared to the research carried out on how women experience the grieving process when facing this type of loss, there is still little known about the psychological impact that this type of death has on men.

The purpose of this study was to summarize and synthesize the existing literature on the way grief is experienced by men who experience the death of a child during the perinatal stage. 

## 2. Materials and Methods

A scoping review was carried out to explore experiences of grief in men who have lost a child during the perinatal stage. The PRISMA methodology and extension guidelines for scoping reviews [13] were followed (see Supplementary Materials (File SI)). The research question used for the scoping review was: How is perinatal grief experienced by men whose children have died during pregnancy or the first month of life, and what is its psychological impact?

### 2.1. Inclusion and Exclusion Criteria

**Figure 1:**
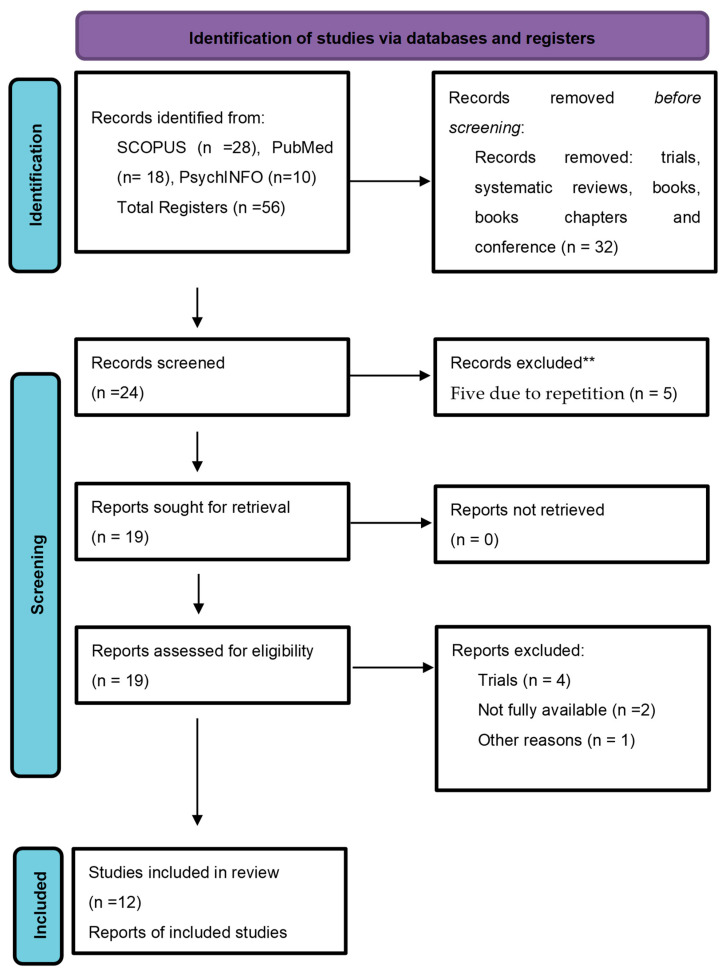
Diagram of the selection of articles. ** In the first reading identical manuscripts were found.

Initially, the selection criteria included every available English research article related to perinatal grief in parents and published between January 2019 and May 2022 (this time period was determined in order to obtain the most current investigations). This search produced 56 papers. The criteria were refined as our familiarity with the available literature increased, and we selected 24 articles that met the following inclusion criteria: articles that referred to the psychological impact and experience of perinatal grief in men; were written in English; were carried out with a quantitative, qualitative, or mixed methodology; and were published during the designated period. Simple and systematic reviews, meta-analyses, conference abstracts, books or book chapters, texts from informative journals, and degree theses were excluded. In a second review of the same 24 articles, 12 were deleted, as follows: 5 articles due to repetition, 4 that were trials, 2 whose texts were not fully available, 1 whose sample comprised health care providers, and 1 in which there was only one man in the sample. A total of 12 articles remained for full-text analysis (see Figure 1).

### 2.2. Search Strategy

We conducted a systematic search for original articles in three databases (Scopus, Pubmed, and PsychINFO). In the initial search, “perinatal loss” AND “fathers” were the two terms used as titles or keywords. To focus on studies about the experience of grief and its psychological impact, the terms “perinatal grief” OR “psychological impact” were used and, in turn, combined with AND to reduce the search scope.

### 2.3. Data Extraction and Charting Process

Once the search was finished, the data collection for each article was carried out by the first author and independently reviewed by the other three co-authors. The obtained data were organized in a form that accounted for the main characteristics of each article: title, year of publication, country of study, characteristics and sample size, type and time of loss, and type of study. 

Subsequently, a thorough reading of each article was performed, followed by a qualitative analysis in which the objective and main results of each study were identified and extracted. Once the information was organized, three common themes were identified: the experience of perinatal grief from the paternal perspective, the psychological impact of perinatal death on men, and the care needs that this population requires. 

## 3. Results

### 3.1. Characteristics of the Studies

As shown in Table 1, the studies examined different samples, with a total of 659 participants, of whom 67.83% (447) were men and the remaining 32.1% (188) were women. In terms of age, all study participants were older than 18 years, and only five studies mentioned the average age. Regarding the type of loss, most of the studies (8) were on fetal deaths, two examined neonatal death, and two included both types. Similarly, the vast majority of the studies (8) took into account the time elapsed since the loss, reporting a very wide range that spanned from 2 months to 20 years. Four articles did not include any data about the time elapsed. Nine studies used qualitative methodologies by means of in-depth semi-structured interviews, all of which were recorded and transcribed, as their data collection technique. Two studies were quantitative, and one used a mixed methodology. Both the quantitative and the mixed studies applied sociodemographic questionnaires related to perinatal loss. The measurement of perinatal grief was obtained using PGS-33 [14]. In addition, the authors used other scales according to the objective of each study.

#### Relevant Topics

The qualitative analysis of the articles allowed for the identification of the following common themes (see Table 2). Their descriptions can be found in Supplementary File S2.

*Experience and reactions to loss.* One of the main themes found in most of the articles was the impact on and response of men when they learned of the death of their baby, regardless of whether it occurred during the pregnancy or the neonatal period [5,9,11,15,17,18,19,20,22,23]. Some frequently reported reactions included shock caused by the unexpected news; pain caused by the loss; feelings of sadness, helplessness, and hopelessness; and a feeling of emptiness [5,19,23]. Most fathers agreed that they felt compelled to repress and control these feelings in order to meet the social expectations of them as men.

*Role of the fathers during the grieving process*. Regarding men’s attitude during the grieving process, all of the studies found that the fathers assumed the duty of supporting their partners during it [5,23]. They also perceived a difference in the way men express their pain when compared to women, as well as a difference in its intensity, with men stating that their grief was less intense than that of women [16,17,18]. They also reported silencing their own grieving process and, rather, experiencing it as unauthorized grief.

*Impact of perinatal death*. These findings show that men perceive that the death of their children has impacts on different areas of their lives. One of these areas is family dynamics, as engaging in a struggle between maintaining the protective role that has been socially assigned to men and succumbing to the pain caused by the loss affects the attention and care they provide to other children and family members. Another affected area reported by fathers was work, since their altered emotional state lowered their job performance. Some of the studies noted that the men’s relationships with their partners were also affected by gender differences found in the expression and type of perinatal grief [9,16,18,21].

Similarly, men’s social environments were also affected, as they tended to isolate themselves from their surroundings in order to avoid unfortunate comments made by family and friends and caused by the non-acknowledgement of grief and the minimization of the men’s loss. The quantitative studies showed that the men who received greater social and medical support had lower scores regarding the intensity of their grief, whereas those who showed marital dissatisfaction and lesser attachment to the baby during the pregnancy obtained higher-intensity scores [16] (see Table 2).

*Needs of the fathers during the grieving process*. Table 2 illustrates that another important theme related to the experience of perinatal grief in men is the acknowledgement of their needs when facing the deaths of their children. One of these is the need to be acknowledged as fathers, even if the death of the baby prevents them from carrying out the functions socially assigned to the paternal role. Another need is related to the care men receive from the health professionals who look after their partners. Most of the studies found that fathers felt dissatisfied with their health providers because they sensed that all the attention was focused on their wives, thus minimizing their own pain. Moreover, it is noted that the fathers needed to spend time with their deceased babies in order to be able to say goodbye and strengthen their affective bond, and they also needed to receive more information about what happened, the cause of the death, and grief in order to better process the experience [19,20,22].

*Coping with grief*. Regarding this subject, it was found that among the strategies men use to cope with perinatal loss, the ones most used are related to becoming more involved in spiritual and religious activities, as well as out-of-home and work-related activities. In two of the reviewed studies, it was noted that men tended to use instrumental grief—which is based on problem solving—as a coping strategy, while very few used intuitive or emotional grief [11,17,21,23].

## 4. Discussion

Despite the fact that only three databases were consulted for this study (it was not possible to consult other equally important ones), the results of this review, in accordance with other similar works [24,25,26], primarily highlight the shortage of studies referring to the impact and characteristics of perinatal loss in fathers when compared to the amount of research conducted about grief in mothers; over a four-year period, only 12 studies on the subject were found. In addition, there is an even greater shortage of quantitative research including predictive analyses or risk factors for the development of grief, which would allow for the generalization of the results. To some extent, It seems that fathers who have experienced perinatal loss have also been rendered invisible in this field of research. Another aspect of interest is the fact that most studies were carried out in European populations and, thus, perinatal loss experienced by men is a rarely explored phenomenon in America and, in particular, in Latin American populations, where forming a family and becoming parents are highly valued aspects of society for both women and men. The present review only found one study carried out in Colombia.

The second important finding of this review is that there is a lack of acknowledgement from both society and health professionals of perinatal death and grief as traumatic and highly complicated experiences for men. This is heightened by the need to recognize the deceased baby as an irreplaceable individual who is a member of the family, since according to Cassidy [2], failing to do turns the experience into marginalized grief, thus hindering the validity of the loss, which is an important element for the normal development of grief.

On the other hand, in accordance with what has been noted in other research [27,28], it cannot be denied that the loss of a child during the perinatal stage provokes emotional alterations in men similar to those experienced by women, and these affect almost every area of their lives. Nevertheless, the analyses of articles carried out in this review highlight that the panorama of emotional impacts created by perinatal death is nuanced by gender stigmas that complicate the objective understanding of this phenomenon and have an impact on the multidisciplinary care provided to the parents. This situation contributes to the belief that fathers whose babies die in the perinatal stage experience less complicated grief than their wives and, therefore, do not require further support. In this sense, it must be highlighted that men need equal treatment and that the differentiated treatment of women must be changed, not only in terms of medical and hospital attention but also when it comes to the attitudes of different social groups. 

Additionally, it was observed that the fathers in the samples included in the 12 reviewed articles consistently rated the attention and care they received from health professionals at their respective hospitals as unsatisfactory, since some of their needs were not adequately met. These needs included being allowed to spend more time with their deceased babies, receiving clear and precise information on the causes of death and the clinical procedures to which their partners were subjected, and receiving the proper psychological and emotional support that they required in those moments, which would have allowed them to recognize their right to express their grief. It is very important that health professionals provide both women and men with adequate care and attention to cope with grief; nevertheless, as pointed out by Fernández-Basanta [27], most of the time, the attention is only directed towards the medical aspects of the situation, leaving the psychological sphere unaddressed. In this regard, the lack of research related to psychological interventions for fathers experiencing perinatal loss must also be highlighted, as up until now, most studies have only aimed to explore emotional reactions and their impacts. Furthermore, the results of this review assist us in identifying future lines of research, such as perinatal grief in Latin American fathers, since the studies in this region are scarce, as well as the impact of perinatal loss on couples’ dynamics and relationships, and the influences of other variables (such as marital satisfaction, communication styles, problem-solving strategies, types of coping, and attitudes toward fatherhood, amongst others) in the development of perinatal grief. An additional line of research that is truly worth exploring would be how perinatal grief is experienced by homoparental families, as this population has been studied even less.

## 5. Conclusions and Limitations

Amidst the common experiences found in the articles included in this review, the following stand out: the importance of acknowledging the fetus or neonate as an irreplaceable and unique individual who has a place in the family; the acknowledgement of the validity of men’s loss and perinatal grief by their social circles, family, and health professionals; the opportunity for fathers to spend more time with their deceased babies, which may allow them to strengthen their affective bond and create memories that help them assimilate the experience; and, finally, the availability of emotional care and support for grieving fathers, as well as the ability to receive full and timely information about the death of a child and its emotional impact.

It is important to underline that a limitation of this study is the fact that we only obtained access to three databases; therefore, the results may reflect only some of the important aspects of the impacts of perinatal death and paternal grief.

## Figures and Tables

**Table 1:** Characteristics of the studies.

Study	Country	Type of Study	Sample	Type of Death	Time of Loss
Lizcano et al., (2019) [5]	Colombia	Qualitative	15 Spanish-speaking men over 18 years (average age not reported)	Gestational	No more than one year
Fernández-Solá et al., (2020) [9]	Spain	Qualitative	8 fathers and 13 mothers of legal age (average age not reported)	Gestational	3 months to 5 years
Das et al., (2021) [15]	India	Mixed exploratory	49 fathers and 50 mothers (average age not reported).	Gestational	6 to 9 months
Obst et al., (2021) [16]	Australia	Quantitative	228 heterosexual males over 18 years	Gestational and neonatal	The past 20 years
Azeez et al., (2022) [11]	Australia	Qualitative exploratory	10 men over the age of 18; age ranged from 31 to 42 years (M = 32, SD = 3.4).	Neonatal	From 1 to 12 years
Tanacıoglu-Aydın & Erdur-Baker (2022) [17]	Turkey	Phenomenological–qualitative	10 couples, a total of 20 subjects (10 men and 10 women) over the age of 18	Gestational	4 months to 6 years
King et al., (2019) [18]	USA	Qualitative	8 couples (8 men and 8 women), mean age for women = 29.88, SD = 6.06; for men = 31.63, SD = 6.65	Gestational	3 to 6.5 years
Camacho et al., (2020) [19]	Spain	Qualitative	21 subjects (13 mothers and 8 fathers), mean age of 35.6 years.	Gestational and neonatal	Not mentioned
Norton et al., (2020) [20]	United kingdom	Qualitative	7 participants (4 mothers and 3 fathers)	Neonatal	Not mentioned
Faleschini et al., (2021) [21]	Canada	Quantitative	92 dyads (father and mother) = 184. Women had a mean age = 31.19, SD = 4.24; the mean age for men = 33.17, SD = 4.21.	Gestational	Not mentioned
Farrales et al., (2020) [22]	Canada	Qualitative	15 women and 12 fathers over 19 years old. The median age was 39 years.	Gestational	2 months to 20 years
Martínez-Serrano et al., (2019) [23]	Spain	Qualitative	7 mothers and 4 fathers over 18 years old.	Gestational	> 18 months

**Table 2:** Main findings of the articles.

Study	Objective	Identified Themes
Lizcano et al., (2019) [5]	To understand and describe the meaning of perinatal death in a sample of parents from northeastern Colombia.	(1) The experience of loss. (2) The irreparable loss. (3) Overcoming loss.
Fernández-Solá et al., (2020) [9]	To explore the social and psychological impacts of infant death on the parents and their families in northern India.	(1) The impact on family dynamics. (2) The impact on the social environment.
Das et al., (2021) [15]	To document the grief and coping experiences of Indian parents after stillbirth and neonatal death.	(1) Anticipation and expression of grief. (2) Impact of grief. (3) Coping mechanisms. (4) Socio-cultural practices and norms.
Obst et al., (2021) [16]	To determine factors associated with grief intensity afterpregnancy loss and neonatal death, as well as factors associated with intuitive and instrumental grief styles.	(1) Grief intensity is related to gestational age. (2) Social support and the bond with the baby during pregnancy are related to less intense mourning.
Azeez et al., (2022) [11]	To explore the grieving experiences of fathers following neonatal death.	(1) Grief as a complicated experience. (2) The multidimensionality of grief. (3) Sense of injustice.
Tanacıoglu-Aydın & Erdur-Baker (2022) [17]	To identify the experiences of pregnancy loss among couples.	(1) The sociocultural context before the pregnancy. (2) The sociocultural context after the loss.
King et al., (2019) [18]	To understand the hospital experiences of fetal death for parents, particularly men, and to understand how couples experienced it together.	(1) Hospital care. (2) Grief and loss. (3) The relationships with their partner and family. (4) Long-term impacts.
Camacho et al., (2020) [19]	To describe and understand the experiences of parents in relation to professional and social support after fetal and neonatal death.	(1) Unauthorized grief. (2) Lack of social acknowledgement. (3) Socially, the loss is minimized.
Norton et al., (2020) [20]	To discover the experiences of parents whose children died in the perinatal period and who used cold cribs for preservation.	(1) Having space and time to be able to adjust to the loss. (2) Being able to care for the baby for a while. (3) Being able to spend family time with the baby. (4) Having the baby close. (5) Creating memories. (6) Social perception of being able to spend time with the deceased baby.
Faleschini et al., (2021) [21]	To examine associations between perinatal losses, psychological symptoms, and parental stress in mothers and fathers six months after the birth of a subsequent healthy child.	(1) Various losses increase psychological symptoms. (2) Risks may be influenced by gender stigmas, socialization, and biological and physiological differences between men and women. (3) Men reported fewer symptoms than women.
Farrales et al., (2020) [22]	To explore the experiences of bereaved parents during their interactions with healthcare providers during and after the stillbirth of an infant.	(1) The acknowledgement of the baby as an irreplaceable individual. (2) The acknowledgement of fathers’ paternity and grief. (3) The acknowledgement of traumatic grief. (4) The acknowledgement of the need for specialized support.
Martínez-Serrano et al., (2019) [23]	To explore the experiences of both mothers and fathers regarding care received during childbirth in cases of stillbirth.	(1) Grief denial. (2) The paradox of life and death. (3) Guilt. (4) The experience and overcoming of loss.

## Data Availability

https://docs.google.com/spreadsheets/d/1s2GdDTNF4ZLXcUGq8Rq445dLwmXik33B/edit?usp=sharing&ouid=101139543258566476287&rtpof=true&sd=true accessed on 17 January 2023.

## Supplementary Materials

The following supporting information can be downloaded at https://www.mdpi.com/article/10.3390/ijerph20064886/s1. File S1—Scoping Review (PRISMA-ScR) Checklist, File S2—Main Findings.
