# Supplementary material for: Paternal Experiences of Perinatal Loss—A Scoping Review
Source: Int J Environ Res Public Health. 2023 Mar 10;20(6):4886. doi: 10.3390/ijerph20064886 (PMC10049302; doi:10.3390/ijerph20064886)
Supplement: Supplementary file 1 [file ijerph-20-04886-s001.zip › ijerph-2199434-supplementary.pdf]

S1. Scoping Reviews (PRISMA-ScR) Checklist.

SECTION	ITEM	PRISMA-ScR CHECKLIST ITEM	REPORTED ON PAGE #
**TITLE**			
Title	1	The title mentions that it is a scoping review	1
**ABSTRACT**			
Structured summary	2	The abstract of this manuscript is structured as follows: background, objectives, eligibility criteria, sources of evidence, charting methods, results, and conclusions that relate to the review questions and objectives.	1
**INTRODUCTION**			
Rationale	3	The importance of the study on perinatal bereavement in both mothers and fathers is described and the research on this type of bereavement in men is summarized, which serves as a context for the objective of this review.	1-2
Objectives	4	The objective is clearly described	1-2
**METHODS**			
Protocol and registration	5	Even though in the methodology for scoping review it is mentioned that the registration of the protocol is not necessary. This protocol has been registered at https://osf.io/registries?view_only= with the following identifier : osf-registrations-9wst7-v1	9
Eligibility criteria	6	For the search, the three institutionally available databases were used. Inclusion and exclusion criteria were established for the studies and the years of publication of the articles were established, with the aim of knowing the most recent ones.	2-3
Information sources*	7	The databases used were: Scopus, Pubmed and PsychINFO, the last search date was September 2022. The terms were used: "perinatal loss", "fathers", "perinatal grief", "psychological impact" with the Boolean operators " AND" "OR"	2
Search	8	It was not included in the manuscript.	
Selection of sources of evidence	9	Articles that met the inclusion criteria were selected, leaving systematic reviews, meta-analyses, and gray literature out of the analysis.	2-3
Data charting process	10	To organize and graph the search data, excel sheets were used and a flowchart of the search steps was made.	7-8

Data items	11	They were defined but this information was not included in the manuscript.	
Critical appraisal of individual sources of evidence§	12	An analysis of the content of each one of the articles that were considered for the analysis was carried out, the data was organized in excel tables by categories. The author, country, year of publication, type of study, objective, methodology, as well as main findings were recorded.	3
Synthesis of results	13	Identification and analysis of main themes common to all articles	

SECTION	ITEM	PRISMA-ScR CHECKLIST ITEM	REPORTED ON PAGE #
**RESULTS**			
Selection of sources of evidence	14	It was described by means of a flowchart that shows the number of articles found from the first search to the final search.	7-8
Characteristics of sources of evidence	15	This information is contained in table 2 of the article and supplementary file 2	3-7
Critical appraisal within sources of evidence	16	An analysis of what was stated in Table 2 was carried out.	3-6
Results of individual sources of evidence	17	They are presented throughout the results section.	3
Synthesis of results	18	They do appear in the manuscript	3
**DISCUSSION**			
Summary of evidence	19	The results were linked to the objective of the review and the initial question.	8-9
Limitations	20	Limitations were pointed out, mainly the number of databases consulted.	
Conclusions	21	A general interpretation of the results linked to the objective is exposed.	9
**FUNDING**			
Funding	22	N/A	

JBI = Joanna Briggs Institute; PRISMA-ScR = Preferred Reporting Items for Systematic reviews and Meta-Analysesextension for Scoping Reviews.

S2. Main findings of the articles.

**Study**	**Objective**	**Findings**
Experience of Perinatal Death from the Father's Perspective	To understand and describe the meaning of perinatal death in a sample of parents from northeastern Colombia	Three themes are identified**: 1) The experience of loss**, which included sadness, pain and loneliness as well as feeling little empathy from the health personnel. **2) The irreparable loss** after a normal pregnancy is experienced with weakness and hopelessness, and with the commitment of supporting their partner in grief. **3) The overcoming of loss,** which involved finding spiritual and mental harmony by remembering their child and finding meaning in his/her death.
Impact of Perinatal Death on the Social and Family Context of the Parents	To explore the social, emotional and psychological impact of infant death on the parents and their families in northern India.	It yielded two main themes: **1) The impact on family dynamics**, described as a struggle between preserving the socially assigned protective role and succumbing to the pain of the loss, which affects the care and attention to other children and weakens the couple's bond. **2) The impact on the social environment.** Men perform poorly in their work life due to their emotional state and not having enough resting days to process their pain. They are also affected in their social environment, since they tend to isolate themselves to avoid unfortunate comments and the non-acknowledgement of their grief by extended family and friends.
Grief Reaction and Psychosocial Impacts of Child Death and Stillbirth on Bereaved North Indian Parents: A [illegible]	To document the grief and coping experiences of Indian parents after stillbirth and neonatal death.	Four categories were obtained: **Anticipation and expression of grief, impact of grief, coping mechanisms** and **socio-cultural practices and norms**. Considering these themes, the following reactions to loss were observed in fathers: shock, sadness, pain, mixed emotions, impotence, and emptiness, with no manifestation while also showing restraint. In addition, they felt dissatisfied with the health-care providers. Some expressed guilt and having problems with their partner. As for how to deal with the loss, it was observed that they became more involved in spiritual and religious activities. Nine months after the loss, grief persisted in those fathers and mothers who suffered a death compared to those who suffered a neonatal death.
Factors Contributing to Men's Grief Following Pregnancy Loss and Neonatal Death: Further Development of an Emerging Model in an Australian Sample	To determine factors associated with grief intensity after pregnancy loss and neonatal death, as well as factors associated with intuitive and instrumental grief styles.	By using linear regression analysis, the study found that the men who had lost their babies in the later stages of pregnancy had higher grieving scores. They also experienced marital dissatisfaction and felt their grieving was seldom acknowledged by others. The grieving scores also differed depending on the type of grief: the men who had higher scores of social support, sensed a greater attachment to their baby during pregnancy, and felt their grief was acknowledged by their partner, were the ones whose instrumental grief score was lower.
Overwhelming and Unjust: A Qualitative Study of Fathers' Experiences of Grief Following Neonatal Death	To explore the grieving experiences of fathers following neonatal death.	Three main themes were generated: **Grief as a complicated experience, the multidimensionality of grief,** and **a sense of injustice.** In general, grief after neonatal death was described as a complicated, highly emotional and absorbing experience. The grief experience was reported to be influenced by a reduced ability to process new information, feelings of anger, and physical symptoms. Fathers used a wide range of strategies in response to their acute grief, and the death of their baby often had long-term impacts on their identity.
Pregnancy Loss Experiences of Couples in a Phenomenological Study: Gender Differences Within the Turkish Sociocultural Context	To identify the experiences of pregnancy loss in couples.	Regarding socio-cultural factors, two themes emerged: **The sociocultural context before the pregnancy** and **the sociocultural context after the loss**. Before a pregnancy there is pressure to have a child and great expectation for the gender of the baby. After the loss, the men stated that their wives reacted with greater emotionality for they feel that the emotional bonds are stronger in them because they carry and give birth to the baby. They also perceive a difference in the way they express their grief: Since they're affected by the social opinion and expectation of them as the ones who provide support and strength to their partners, they must repress their grief. Another aspect they consider quite important to feel relieved is social support, particularly from their friends. It was observed that men use work, activities outside the home and instrumental grief as coping strategies.

Pain Without Reward: A Phenomenological Exploration of Stillbirth for Couples and their Hospital Encounter	To understand the hospital experience of fetal death in parents, particularly men, as well as understanding how they experienced it together.	The main themes were**: hospital care, grief and loss, the relationship with their partner and family,** and **long-term impacts**. The following were found to be quite important: the care provided in hospitals, receiving clear and accurate information, and the notification of the loss as a highly impactful event. It helped them to be able to see their baby and spend time with him/her. The fathers stated that their role was supportive and their grief was different and less intense than that of their wives.
Parents' Experiences About Support Following Stillbirth and Neonatal Death	To describe and understand the experiences of parents in relation to professional and social support after fetal and neonatal death.	The main findings mention that grief is avoided in this type of loss and that the parents silence their pain, which results in unauthorized grief. The parents' environment minimizes their loss, religious rites become absent and this contributes to a lack of social acknowledgement.
Parents' Experience of Using "Cold" Facilities at a Children's Hospice After the Death of their Baby: A Qualitative Study	To know the experiences of moms and dads whose children died in the perinatal period and used cold cribs for preservation.	Six themes were identified in their grief experience: **having space and time to be able to adjust to the loss; being able to care for the baby for a while; being able to spend family time with the baby; having the baby close; creating memories; and the social perception of being able to spend time with the deceased baby**. The positive experience of being able to create memories and bonds with the baby (thus validating his/her existence) and going through slow detachment corroborates the importance of continuing with the affective ties towards the baby during the grieving process.
History of Perinatal Loss: A Study of Psychological Outcomes in Mothers and Fathers After Subsequent Healthy Birth	To examine associations between perinatal losses, psychological symptoms, and parental stress in mothers and fathers six months after the birth of a subsequent healthy child.	Psychological symptoms increased as losses increased, and men reported fewer symptoms than women. However, both are at risk of psychological impairment even after the birth of a healthy child. Depression and anxiety remain for up to six months after the birth of a new child. Risks may be influenced by gender stigmas, socialization, and biological and physiological differences between men and women. It is important to look for differences between men and women in terms of coping strategies.
What Bereaved Parents Want Health Care Providers to Know When Their Babies Are Stillborn: A Community-based Participatory Study	To explore the experiences of bereaved parents during their interaction with healthcare providers during and after the stillbirth of an infant.	One main theme is identified: **The acknowledgement of the baby as an irreplaceable individual**; and three sub-themes**: The acknowledgement of the fathers' paternity and grief, the acknowledgement of traumatic grief and the acknowledgement of the need for specialized support**. No differences were found between mothers and fathers regarding the three themes; men and women talked about traumatic grief and the need for acknowledgement and support in a similar way. The main theme focused on the parents' desire for acknowledgement of their babies as irreplaceable individuals and the acknowledgement of parental ties, which in turn validate the pain of the loss. Parents perceive the need to spend time with their baby to elaborate rituals.

The Experience of Mothers and Fathers in Cases of Stillbirth in Spain: A Qualitative Study	To explore the experience of both mothers and fathers regarding care received during childbirth in cases of stillbirth.	Four main themes were identified: 1) **GRIEF DENIAL:** Most participants were unaware that stillbirth could occur. The parents' suffering is underestimated and they're expected to recover immediately for it is perceived as a minor loss. In the case of fathers, this denial also has an aspect of will for the grief itself is postponed to support their partner. Women become their main concern and they are not aware this expropriation will have consequences. Families also focus on women and rarely pay attention to the father. In short, the paternal grief is relegated. Another form of grief denial comes from health professionals who made them feel misunderstood about their sense of loss and grief. 2) **THE PARADOX OF LIFE AND DEATH:** Fetal death implies a paradoxical experience that does not occur in other circumstances of life: that of childbirth, an act linked to life and the death of the child at the same time. For parents, their lost child is present every day for the rest of their lives, and they feel the need to express about the event, to be able to speak naturally about their child in the same way they do about their living children, with no taboos. 3) **GUILT:** Guilt appeared universally in all participants, regardless of whether they were mothers or fathers. This feeling also extended to later moments, actions or omissions that occurred during childbirth and immediately after, such as refusing to see or hold the child, having been afraid of seeing it, not keeping memories, or not taking care of the body. 4) **THE EXPERIENCE AND OVERCOMING OF LOSS:** The moment of diagnosis was perceived as mishandled and tactless. The sense of unreality was accompanied by disbelief, the denial of death, attempts to escape the situation, or the need to verify the death with hopes for a medical error. Parents who see and/or hold their child believe it helps make the loss real, but parents didn't always get the counseling they needed to help them make a decision. Another important decision relates to preserving memories of the newborn, such as photographs, fingerprints or a small strand of hair, all of which were acknowledged as positive. The support received was especially relevant to the father.
